# Institutional Delivery and Satisfaction among Indigenous and Poor Women in Guatemala, Mexico, and Panama

**DOI:** 10.1371/journal.pone.0154388

**Published:** 2016-04-27

**Authors:** Danny V. Colombara, Bernardo Hernández, Alexandra Schaefer, Nicholas Zyznieuski, Miranda F. Bryant, Sima S. Desai, Marielle C. Gagnier, Casey K. Johanns, Claire R. McNellan, Erin B. Palmisano, Diego Ríos-Zertuche, Paola Zúñiga-Brenes, Emma Iriarte, Ali H. Mokdad

**Affiliations:** 1 Institute for Health Metrics and Evaluation, 2301 5th Ave, Suite 600, Seattle, Washington, United States of America; 2 Salud Mesoamérica Initiative, Inter-American Development Bank, Calle 50 con Calle Elvira Méndez, Edificio Tower Financial Center (Towerbank), Piso 23, Panamá City, Panamá; Universiti Sains Malaysia, MALAYSIA

## Abstract

Indigenous women in Mesoamerica experience disproportionately high maternal mortality rates and are less likely to have institutional deliveries. Identifying correlates of institutional delivery, and satisfaction with institutional deliveries, may help improve facility utilization and health outcomes in this population. We used baseline surveys from the Salud Mesoamérica Initiative to analyze data from 10,895 indigenous and non-indigenous women in Guatemala and Mexico (Chiapas State) and indigenous women in Panama. We created multivariable Poisson regression models for indigenous (Guatemala, Mexico, Panama) and non-indigenous (Guatemala, Mexico) women to identify correlates of institutional delivery and satisfaction. Compared to their non-indigenous peers, indigenous women were substantially less likely to have an institutional delivery (15.2% vs. 41.5% in Guatemala (P<0.001), 29.1% vs. 73.9% in Mexico (P<0.001), and 70.3% among indigenous Panamanian women). Indigenous women who had at least one antenatal care visit were more than 90% more likely to have an institutional delivery (adjusted risk ratio (aRR) = 1.94, 95% confidence interval (CI): 1.44–2.61), compared to those who had no visits. Indigenous women who were advised to give birth in a health facility (aRR = 1.46, 95% CI: 1.18–1.81), primiparous (aRR = 1.44, 95% CI: 1.24–1.68), informed that she should have a Caesarean section (aRR = 1.41, 95% CI: 1.21–1.63), and had a secondary or higher level of education (aRR = 1.36, 95% CI: 1.04–1.79) also had substantially higher likelihoods of institutional delivery. Satisfaction among indigenous women was associated with being able to be accompanied by a community health worker (aRR = 1.15, 95% CI: 1.05–1.26) and facility staff speaking an indigenous language (aRR = 1.10, 95% CI: 1.02–1.19). Additional effort should be exerted to increase utilization of birthing facilities by indigenous and poor women in the region. Improving access to antenatal care and opportunities for higher-level education may increase institutional delivery rates, and providing culturally adapted services may improve satisfaction.

## Introduction

Over the past 15 years, there have been widespread reductions in the maternal mortality ratio across much of the globe [1]. Despite this improvement, there were nearly 300,000 maternal deaths in 2013 [1], possibly more due to underreporting [2]. Since the majority of maternal, fetal, and neonatal deaths take place during or soon after delivery [1,3,4], institutional delivery with skilled birth attendants is expected to dramatically reduce the number of these deaths [5,6]. Even so, the global epidemic of cesarean sections may also have contributed to excess maternal mortality [7]. For example, between 1990 and 2014 Central American cesarean section rates are estimated to have increased two and a half fold [7]. Nevertheless, there has been a strong push for institutional deliveries throughout Central America. However, greater availability of health facilities alone will not necessarily improve utilization [8] or reduce health inequities.

When compared to non-indigenous women, indigenous women in Latin America are less likely to have an institutional delivery with a skilled birth attendant [9,8,10–12], and indigenous communities may experience maternal mortality rates that are two to three fold greater than the national averages [13]. Indigenous women may be less likely to deliver in a health facility for many reasons. Some studies have reported that costs and challenges due to topography or distance pose formidable obstacles to utilizing health facilities [14,15], while other studies found that these factors have a limited influence on a woman’s decision to seek delivery through the formal medical system [8,16]. Others have noted the impact of intra-household power dynamics, in which decisions regarding utilization of birthing facilities are often made by the husband or his family rather than the parturient woman [13,17,18]. Still others have noted the impact of perceived low quality of available care [14] and previous experiences of coercion regarding family planning [19]. Indigenous women may also have expectations that their births will be “normal” and therefore not need medical attention [20]. In addition, indigenous women may fear cesarean sections, which may be confused with tubal ligations, and defer to traditional birth attendants regarding the decision to seek emergency obstetric care [20]. Furthermore, references by midwives to “obstetric violence” in association with institutional delivery [21] may dissuade some women from seeking an institutional delivery.

In addition to the above, indigenous women may need to cross a cultural divide in order to utilize facilities that often fail to adequately respect indigenous cultures [4,14,22]. Health care staff in the region tend to be non-indigenous and often do not speak indigenous languages, even if their patients are predominantly indigenous [23]. Furthermore, not all health facilities provide culturally adapted services, which include accompaniment by a traditional midwife or family members, linguistically competent facility staff, the ability to consume traditional beverages, and permitting alternative birthing positions [4]. In contrast, indigenous women often experience “strong social and cultural identification” with traditional birth attendants who may also be more flexible in allowing women to choose their birthing position [24,16,25]. In some settings, such as Guatemala, the government has expressly noted a woman’s right to choose her birthing position, including: sitting, kneeling, squatting, standing, lying on her left side, being suspended, and using a vertical bed [26].

Although some previous studies have examined correlates of institutional delivery, few used representative samples, had corresponding health-facility data, or compared indigenous women to non-indigenous women of comparable socio-economic status to identify associations that are unique to indigenous women. We therefore conducted a cross-sectional study to identify correlates of institutional delivery and satisfaction (whether a woman would deliver in the same facility again) among the poorest 20% of women living in Guatemala, Mexico, and Panama using household and health-facility survey data. We also examined reasons why women who delivered in a given facility would not do so again and compared indigenous and non-indigenous women with regard to health system utilization based upon a continuum of care.

## Methods

### Data collection

The Salud Mesoamérica Initiative (SMI) is a results-based aid program that seeks to improve the health of the poorest 20% of women and children living in Belize, Costa Rica, El Salvador, Guatemala, Honduras, Mexico (Chiapas State), Nicaragua, and Panama. The conduct of the SMI baseline health-facility surveys and population-based household surveys has been described previously [27]. In summary, aggregate-level measures were used to identify the poorest quintile of municipalities in each country. However, since it is known to be the poorest state, sampling in Mexico was limited to Chiapas. Localities, which were the primary sampling unit (PSU), were selected within the municipalities with probability proportional to size and averaged approximately 150 households. We conducted a census within selected localities and randomly selected 30 households with women aged 15 to 49 years or children under 5 for in-depth surveying. We also surveyed a sample of public health facilities in the selected municipalities. Interviewers conducted computer-assisted personal interviews (CAPI) in Spanish or, when appropriate, in local indigenous languages.

### Study population

We limited this analysis to the three participating countries with sizeable indigenous populations: Guatemala (surveyed April-August 2013), Mexico (surveyed August 2012-May 2013), and Panama (surveyed April-August 2013). In Guatemala and Mexico, a woman was considered indigenous if an indigenous language was spoken in her household, regardless of whether it was her primary language. The Panamanian surveys were conducted in tribal communities where all surveyed individuals had an indigenous language spoken in their home. To avoid potential bias due to the lack of non-indigenous women, Panamanian data were not included in cross-country analyses.

### Data analysis

We compared the characteristics of indigenous and non-indigenous mothers and their closest health facilities within countries using chi-square tests. Sociodemographic characteristics included the women’s age in years (15–24, 25–34, 34–49), highest level of education (none, primary, secondary or higher), and literacy status. Literacy was a binary variable, defined as the ability to completely read a simple sentence regarding child health in Spanish or, when appropriate, in an indigenous language. We also assessed the household’s wealth index, whether the mother was married, whether the mother wanted the pregnancy, whether she was primiparous, and whether her most recent delivery was in a health facility. The wealth index was created by compiling one point for ownership of modes of transportation (scooter, car, or truck), household durable goods (e.g., clothes washer or refrigerator), land, and livestock. The first two quintiles of the asset distribution were categorized as “low,” the third and fourth quintiles were categorized as “medium,” and the fifth quintile was categorized as “high.” Women who reported being married or in a domestic partnership were categorized as married. Those who reported being single, divorced, separated, or widowed were considered not married. We also assessed whether women participated in a conditional cash transfer (CCT) program (*Prospera*–formerly *Oportunidades* [19]–in Mexico, *Mi Familia Progresa* in Guatemala, and *Red de Oportunidades* in Panama). If the most recent birth was in a facility, we also assessed if the woman had a routine or emergency Caesarean section, and the travel time for that delivery (<30 minutes, 30 minutes < 1 hour, 1 <2 hours, 2+ hours).

Assessed closest health facility characteristics included the type of facility (ambulatory, basic, complete), whether the delivery room was adapted for indigenous women, whether the facility as a whole adapts services for indigenous women, and whether the staff speak an indigenous language. Ambulatory facilities provide outpatient services. Basic facilities are staffed with nurses and doctors, have the capacity to attend births, and do not have an operating theater. Complete facilities are hospitals with medical specialists that have the capacity to attend births and have operating theaters. We also examined whether the health facility allowed women to be accompanied in general, and, more specifically, by a community health worker or traditional birth attendant. Finally, we assessed the birthing positions allowable by the facility: in a bed, in a chair, kneeling, sitting, squatting, standing, and using a vertical bed.

We identified the correlates of having an institutional delivery for each country and across Guatemala and Mexico combined. Potential correlates were age, education, literacy, marital status, wealth, CCT program participation, primiparity, and whether the pregnancy was wanted. Other correlates included whether the woman was informed that she should have a Caesarean section, whether she had one or more or four or more antenatal care (ANC) visits, and whether she was advised during an ANC visit to give birth in a health facility or create a transportation plan for an institutional delivery. We also assessed the type of health facility that was closest to the woman and travel time to the closest health facility.

Women who reported having an institutional delivery were asked if they would return to the same facility for a subsequent birth. We defined an affirmative response as “satisfaction” and examined its correlates. Potential correlates included age, education, literacy, marital status, wealth, CCT program participation, the type of facility utilized, travel time to that facility, and whether the woman had a Caesarean section. Additional assessed variables from the household survey included whether the staff spoke the woman’s language, whether she was allowed to be accompanied in general, whether she could wear the clothing of her choice, whether the supplied bed allowed for the birthing position of her choice, whether she was allowed to consume the beverage of her choice, whether she was treated with respect, and whether she was allowed to select the birthing position. Supply-side factors included declaration by the staff that the delivery room was culturally adapted, whether staff could speak an indigenous language, allowing accompaniment in general, and by community health workers and traditional birth attendants in particular, and allowing the various birth positions mentioned above.

We also conducted two exploratory descriptive analyses. In the first, we explored the reasons why some women who gave birth in a facility would not return to that facility. In the second, we assessed utilization of health services along the continuum of care. Measured points along the continuum included having at least one ANC visit, having four or more ANC visits, having an institutional delivery, initiating breastfeeding within one hour of birth, post-natal care (PNC) within 48 hours of delivery, PNC within seven days of delivery, and having a child who is completely vaccinated according to the age-specific vaccination schedule.

### Statistical methods

We assessed the correlates of institutional delivery and satisfaction using Poisson regression with robust variance estimators to estimate the country-specific and cross-country risk ratios (RR) and 95% confidence intervals (CI) [28]. RRs with a p-value <0.10 in univariable analyses were considered candidates for the multivariable models. When using a variance inflation factor (VIF) of >10 as a cut-point, we found no evidence of multi-collinearity among the multivariable candidates for the institutional delivery models. Using the same VIF cut-point, we found evidence of multi-collinearity among some of the satisfaction multivariable model candidates. We therefore omitted accompaniment by a traditional birth attendant from the cross-country analysis, health facility type and accompaniment by a traditional birth attendant from the Guatemala analysis, being allowed to deliver in bed from the Mexico analysis, and being allowed to deliver in a seated or vertical position from the Panama analysis. The remaining multivariable candidates were sorted according to their RRs and added to the model one by one, beginning with the strongest association. Candidates were retained if the corresponding Wald test had a p-value < 0.05. Country was included as a fixed effect in the cross-country analysis and there were no other *a priori* confounders.

Due to a survey programming error, the institutional delivery variable was missing for approximately 20% of Panamanian women. These observations were dropped from the dataset and we conducted a complete case analysis. All other variables were missing less than 5% of the data.

All analyses used two-sided statistical tests, accounted for survey weighting and clustering by PSUs, and were conducted using Stata/IC 13.1 (StataCorp LP, College Station, TX).

### Ethics statement

Written informed consent was obtained from all participants, or their caretakers, or guardians when applicable. The study received institutional review board approval from the University of Washington, partnering data-collection agencies, and the Ministry of Health in each country.

## Results

We analyzed data from a total of 10,895 women: 4,454 in Guatemala (1,081 non-indigenous and 3,373 indigenous), 5,698 in Mexico (1,646 non-indigenous and 4,052 indigenous), and 743 in Panama (all indigenous) (Table 1). Approximately a third of the women in each country were 15–24 years old and 39–48% were 25–34 years old, depending on the country. Compared to their non-indigenous peers, indigenous women in Guatemala and Mexico were more likely to be uneducated, illiterate, have a low wealth index, and to participate in CCT programs (P<0.001). They were also more likely to have wanted their pregnancy. However, they were less likely to have been primiparous (P<0.05) and to have had an institutional delivery (15.2% vs. 41.5% in Guatemala (P<0.001), 29.1% vs. 73.9% in Mexico (P<0.001), and 70.3% in Panama). In Guatemala, more than 60% of women had a closest health facility that was basic or complete level. In Mexico it was between 55% and 60%, and in Panama it was 83.4%. In general, the closest Guatemalan and Panamanian health facilities were more accommodating to indigenous women’s needs than were those in Mexico. Cross-country differences in characteristics of indigenous and non-indigenous women are provided in S1 Table.

**Table 1:** Characteristics of indigenous and non-indigenous women and their closest health facilities in Guatemala, Mexico, and Panama in the Salud Mesoamérica Initiative, 2012–2013.

	Guatemala			Mexico			Panama
	Non-indigenous	Indigenous	P	Non-indigenous	Indigenous	P	Indigenous
	n = 1,081	n = 3,373		n = 1,646	n = 4,052		n = 743
**WOMEN**							
**Age (years)**			0.362			<0.001	
15–24	33.9 (30.2–37.8)	33.4 (31.2–35.6)		37.2 (34.0–40.5)	35.5 (32.9–38.1)		36.3 (31.6–41.3)
25–34	46.0 (41.5–50.5)	43.5 (41.3–45.8)		48.0 (44.5–51.6)	42.9 (40.5–45.3)		39.2 (33.8–44.9)
35–49	20.1 (16.5–24.2)	23.1 (20.9–25.3)		14.8 (12.6–17.2)	21.6 (19.4–24.1)		24.5 (20.4–29.1)
**Education**			<0.001			<0.001	
None	14.1 (10.7–18.4)	40.6 (36.7–44.6)		6.8 (5.2–8.8)	21.9 (18.7–25.6)		15.2 (9.1–24.2)
Primary	58.4 (52.9–63.7)	48.4 (45.5–51.2)		44.5 (38.1–51.2)	56.6 (52.8–60.4)		56.9 (50.6–63.0)
Secondary or higher	27.4 (21.6–34.2)	11.0 (8.6–14.0)		48.7 (42.0–55.4)	21.4 (18.3–24.9)		27.9 (21.8–34.9)
**Literate**	83.4 (79.4–86.8)	57.7 (53.6–61.7)	<0.001	91.2 (88.4–93.3)	70.8 (67.2–74.2)	<0.001	77.8 (69.7–84.2)
**Wealth index**			<0.001			<0.001	
Low	40.0 (32.8–47.6)	62.1 (57.6–66.3)		41.0 (34.6–47.6)	67.5 (63.0–71.6)		42.8 (33.3–52.7)
Medium	29.4 (25.6–33.4)	23.6 (21.0–26.4)		33.6 (30.0–37.4)	23.8 (20.7–27.3)		39.9 (33.2–47.0)
High	30.6 (24.6–37.4)	14.3 (11.9–17.2)		25.4 (21.1–30.2)	8.7 (7.0–10.8)		17.4 (11.2–25.9)
**Conditional cash transfer recipient**	23.7 (19.3–28.6)	39.1 (35.3–43.1)	<0.001	50.3 (42.8–57.8)	75.7 (72.2–78.9)		58.1 (51.9–64.2)
**Married**	83.3 (80.7–86.5)	90.3 (88.8–91.6)	<0.001	91.4 (89.6–93.0)	94.6 (93.6–95.5)	0.001	85.9 (82.7–88.6)
**Wanted the pregnancy**	84.4 (80.5–87.6)	88.9 (86.8–90.8)	0.014	75.1 (71.4–78.5)	82.6 (80.0–84.9)	0.001	61.2 (53.4–68.5)
**Primiparous**	24.9 (21.0–29.2)	19.2 (17.4–21.2)	0.008	24.7 (21.7–28.0)	12.9 (11.5–14.4)	<0.001	15.4 (12.4–18.9)
**Traditional birth attendant assisted in delivery**	0 (-)	0.3 (0.1–1.0)	0.399	2.2 (1.0–5.0)	2.8 (1.3–6.2)	0.663	2.8 (1.4–5.4)
**Woman gave birth in a facility**	41.5 (35.0–48.4)	15.2 (12.4–18.5)	<0.001	73.9 (65.1–81.0)	29.1 (23.9–34.9)	<0.001	70.3 (60.5–78.6)
Caesarean section*	31.3 (25.0–38.4)	28.4 (23.8–33.5)	0.500	35.6 (31.4–40.1)	25.8 (22.8–29.0)	<0.001	3.9 (2.7–5.6)
Emergency C-section*t1s	72.2 (62.2–80.4)	86.7 (79.5–91.7)	0.006	69.5 (63.2–75.2)	78.9 (72.7–84.0)	0.034	65.3 (41.0–83.6)
Delivery travel time*			0.476			<0.001	
<30 min.	15.0 (9.1–23.6)	16.8 (10.6–25.5)		37.5 (30.9–44.7)	18.1 (13.5–23.8)		23.0 (16.6–30.9)
30 min. <1 hr.	19.4 (14.3–25.8)	23.0 (16.9–30.6)		23.5 (19.1–28.7)	22.6 (16.7–29.7)		12.7 (8.0–19.5)
1 hr. to <2 hr.	19.1 (13.5–26.3)	22.8 (16.7–30.2)		15.0 (10.7–20.8)	21.9 (16.7–28.1)		21.5 (15.8–28.5)
> 2 hr.	46.5 (38.6–54.6)	37.4 (29.1–46.6)		23.9 (18.6–30.0)	37.5 (27.3–49.0)		42.9 (33.6–52.7)
**CLOSEST HEALTH FACILITY**							
**Facility type**			0.663			0.899	
ambulatory	33.8 (19.1–52.5)	40.4 (29.8–52.0)		44.1 (29.3–60.0)	40.2 (26.0–56.2)		16.6 (9.0–28.5)
basic	59.9 (42.1–75.4)	53.9 (42.7–64.8)		35.4 (19.9–54.7)	38.5 (23.5–55.9)		83.4 (71.5–91.0)
complete	6.4 (2.3–16.7)	5.7 (2.8–11.3)		20.6 (11.9–33.2)	21.3 (13.1–32.9)		0 (-)
**Delivery room adapted to indigenous populations**†	1.5 (0.4–5.7)	10.4 (4.6–21.9)	<0.001	16.5 (6.0–37.8)	24.6 (9.8–49.5)	0.440	63.1 (39.4–81.9)
**Facility adapts services to the socio-cultural condition of the women**	86.8 (63.3–96.1)	99.4 (97.4–99.8)	<0.001	46.6 (25.5–69.0)	58.4 (37.8–76.4)	0.398	88.7 (70.9–96.2)
**Medical staff speak an indigenous language**	62.4 (39.7–80.7)	99.4 (97.4–99.8)	<0.001	28.2 (13.3–50.0)	48.8 (29.3–68.6)	0.107	88.7 (70.9–96.2)
**Allow accompaniment when coming for delivery**†	90.2 (75.4–96.5)	89.5 (79.7–94.9)	0.876	14.0 (4.0–38.9)	34.0 (15.6–59.0)	0.148	48.0 (26.4–70.4)
**Allow accompaniment by community health worker**†	46.0 (25.1–68.4)	12.3 (4.8–28.1)	0.006	0 (-)	0 (-)		0 (-)
**Allow accompaniment by traditional birth attendant**†	99.9 (99.0–100.0)	95.4 (87.3–98.4)	<0.001	100 (100)	59.4 (19.0–90.2)	0.278	78.3 (40.1–95.1)
**Allowable position: in a bed**†	72.9 (52.0–87.0)	54.0 (40.7–66.8)	0.085	15.4 (5.2–37.5)	30.7 (14.6–53.5)	0.165	11.1 (3.0–33.3)
**Allowable position: in a chair**†	30.7 (15.4–51.8)	11.0 (5.5–20.7)	0.016	0 (-)	0 (-)		40.4 (22.9–60.8)
**Allowable position: on knees**†	47.6 (28.5–67.4)	43.7 (31.1–57.1)	0.728	0 (-)	0.3 (0.0–2.1)	0.416	10.4 (2.6–33.4)
**Allowable position: sitting**†	41.0 (23.2–61.4)	29.8 (19.7–42.4)	0.283	0 (-)	0 (-)		10.4 (2.6–33.4)
**Allowable position: squatting**†	41.4 (23.7–61.7)	30.2 (20.0–42.7)	0.278	0 (-)	0.3 (0.0–2.1)	0.416	10.4 (2.6–33.4)
**Allowable position: standing**†	42.0 (24.1–62.2)	33.0 (22.3–45.8)	0.395	0 (-)	0 (-)		0 (-)
**Allowable position: vertically**†	20.0 (7.9–42.2)	2.5 (0.9–6.8)	<0.001	30.9 (13.9–55.5)	47.2 (27.7–67.6)	0.210	10.4 (2.6–33.4)

*Asked only of women who gave birth in a facility

† Applicable only to women whose closest birth facility had the capacity to attend births, i.e., those that were type basic or complete.

In our cross-country multivariable analysis, we found that non-indigenous women who had at least one ANC visit were 40% more likely to have an institutional delivery compared to those that did not have an ANC visit (adjusted RR (aRR) = 1.40, 95% CI: 1.09–1.81) (Table 2). Among non-indigenous women, we also found increased likelihood for institutional delivery among those who were advised to give birth in a facility (aRR = 1.13, 95% CI: 1.02–1.26) and those having a high wealth index (aRR = 1.13, 95% CI: 1.02–1.26). Non-indigenous women who participated in CCT programs were less likely to have an institutional delivery (aRR = 0.81, 95% CI: 0.72–0.92). Among indigenous women, those who had at least one ANC visit were nearly twice as likely to have an institutional delivery compared to those with no ANC visits (aRR = 1.94, 95% CI: 1.44–2.61). Being advised to give birth in a health facility (aRR = 1.46, 95% CI: 1.18–1.81), being primiparous (aRR = 1.44, 95% CI: 1.24–1.68), being informed that she should have a Caesarean section (aRR = 1.41, 95% CI: 1.21–1.63), and having a secondary or higher level of education (aRR = 1.36, 95% CI: 1.04–1.79) were also associated with increased likelihood for institutional delivery among indigenous women. Indigenous women who were CCT recipients were 35% less likely to have an institutional delivery (aRR = 0.65, 95% CI: 0.55–0.77), and those who were married (aRR = 0.77, 95% CI: 0.63–0.94) and wanted their pregnancy (aRR = 0.81, 95% CI: 0.71–0.92) were also less likely to deliver in a facility. In Guatemala, non-indigenous women whose closest health facility level was complete were more likely to have delivered in a health facility (aRR = 1.80, 95% CI: 1.16–2.79) (S2 Table). Compared to their rural counterparts, urban indigenous Guatemalan women were also more likely to have had an institutional birth (aRR = 1.58, 95% CI: 1.18–2.12). In Mexico the pattern of associations was largely the same as in the cross-country analysis (S3 Table). The Panama-specific analysis did not reveal any additional significant associations (S4 Table).

**Table 2:** Correlates of institutional delivery across Guatemalan and Mexican women in the Salud Mesoamérica Initiative, 2011–2013.

	Univariable	Non-indigenous Multivariable	Indigenous Multivariable
	n = 10,895	n = 2,616	n = 7,671
	RR (95% CI)	aRR (95% CI)	aRR (95% CI)
**Age (years)**			
15–24	1.00		
25–34	0.93 (0.84–1.03)		
35–49	0.85 (0.74–0.98)		
**Education**			
None	1.00	1.00	1.00
Primary	1.50 (1.26–1.79)	0.96 (0.77–1.21)	0.98 (0.74–1.32)
Secondary or higher	2.96 (2.46–3.56)	1.05 (0.84–1.32)	1.36 (1.04–1.79)
**Literate**	2.09 (1.78–2.45)	1.24 (0.98–1.57)	1.13 (0.90–1.43)
**Indigenous**	0.39 (0.32–0.47)		
**Married**	0.79 (0.69–0.91)		0.77 (0.63–0.94)
**Urban**	2.15 (1.71–2.70)	1.17 (0.96–1.44)	
**Wealth index**			
Low	1.00	1.00	
Medium	1.45 (1.24–1.69)	1.02 (0.91–1.14)	
High	1.98 (1.68–2.32)	1.12 (1.01–1.25)	
**Conditional cash transfer recipient**	0.57 (0.50–0.64)	0.81 (0.72–0.92)	0.65 (0.55–0.77)
**Wanted the pregnancy**	0.74 (0.67–0.83)		0.81 (0.71–0.92)
**Primiparous**	1.69 (1.51–1.89)		1.44 (1.24–1.68)
**≥1 skilled antenatal care visit**	3.31 (2.69–4.06)	1.40 (1.09–1.81)	1.94 (1.44–2.61)
**≥4 skilled antenatal care visits**	2.55 (2.18–2.99)	1.09 (0.96–1.25)	1.42 (1.18–1.71)
**Advised to give birth in a health facility**	2.33 (2.04–2.66)	1.13 (1.02–1.26)	1.46 (1.18–1.81)
**Advised to create a transportation plan**	1.66 (1.43–1.92)		
**Informed that should have a C-section**	2.14 (1.90–2.41)		1.41 (1.21–1.63)
**Closest health facility type**			
Ambulatory	1.00		
Basic	0.88 (0.64–1.23)		
Complete	1.02 (0.71–1.47)		
**Travel time to closest delivery facility**			
<30 min.	1.00		
30 min. <1 hr.	0.87 (0.70–1.09)		
1 hr. to <2 hr.	0.87 (0.67–1.13)		
> 2 hr.	0.93 (0.57–1.53)		
**Country**			
Guatemala	1.00	1.00	1.00
Mexico	1.99 (1.59–2.48)	1.35 (1.09–1.68)	1.28 (0.96–1.71)

Travel time to the closest facility was not associated with institutional birth in the cross-country analysis (Table 2) or the country specific analyses (S2, S3, and S4 Tables). In order to further explore this potential association, we limited the analysis to women whose closest facility was basic or complete. In this exploratory univariable analyses, travel time to the closest facility was only significantly associated with institutional birth in Guatemala. Guatemalan women 30 minutes to one hour from a birthing facility had a 33% reduced probability of having an institutional birth (RR = 0.67, 95% CI: 0.48–0.92), those one to two hours away had a 35% reduced probability (RR = 0.65, 95% CI: 0.43–0.97), and those more than two hours away had a 47% reduced probability (RR = 0.53, 95% CI: 0.34–0.81).

Overall, 87.1% of the 2,260 indigenous women and 85.8% of the 1,681 non-indigenous women who had an institutional delivery said they would return for a subsequent delivery. In Guatemala the respective percentages were 77.7% and 80.1%, and in Mexico they were 87.2% and 85.8%. In Panama, which was entirely indigenous, it was 88.1%.

The cross-country analysis of correlates of satisfaction found that the strongest correlate of satisfaction among non-indigenous women was having been treated with respect in the birthing facility (aRR = 2.70, 95% CI: 1.57–4.63), followed by delivering in a facility that allows accompaniment by a community health worker (aRR = 1.20, 95% CI: 1.10–1.32) (Table 3). For indigenous women, the strongest predictor of satisfaction was being allowed to be accompanied by a community health worker (aRR = 1.15, 95% CI: 1.05–1.26), followed by facility staff speaking an indigenous language (aRR = 1.10, 95% CI: 1.02–1.19). In Guatemala, non-indigenous women who delivered in a facility that allowed delivery in a bed (aRR = 1.26, 95% CI: 1.04–1.53) and who were supplied a bed that allowed for the birthing position of choice (aRR = 1.21, 95% CI: 1.03–1.42) were more likely to be satisfied (S5 Table). Being treated with respect was strongly associated with satisfaction among indigenous Guatemalan women (aRR = 1.88, 95% CI: 1.08–3.25). In Mexico, the results were similar to the cross-country analysis (S6 Table). In Panama, indigenous women who were allowed to wear the clothing of their choice (aRR = 1.15, 95% CI: 1.04–1.27), who were allowed to deliver in a bed (aRR = 1.14, 95% CI: 1.07–1.21), and those who were allowed to deliver in a kneeling position (aRR = 1.05, 95% CI: 1.00–1.10) were more likely to be satisfied (S7 Table).

**Table 3:** Correlates of satisfaction among women who gave birth in a health facility in Guatemala and Mexico in the Salud Mesoamérica Initiative, 2011–2013.

	Univariable	Non-indigenous Multivariable	Indigenous Multivariable
	n = 1,570	n = 533	n = 915
	RR (95% CI)	aRR (95% CI)	aRR (95% CI)
**HOUSEHOLD SURVEY**			
**Age (years)**			
15–24	1.00		
25–34	0.96 (0.91–1.01)		
35–49	0.97 (0.91–1.03)		
**Education**			
None	1.00		1.00
Primary	0.97 (0.91–1.03)		0.93 (0.87–1.00)
Secondary or higher	0.94 (0.88–1.00)		0.87 (0.78–0.97)
**Literate**	0.96 (0.91–1.02)		
**Indigenous**	1.02 (0.98–1.05)		
**Married**	1.05 (0.97–1.13)		
**Urban**	0.99 (0.95–1.03)		
**Wealth index**			
Low	1.00		
Medium	0.96 (0.92–1.01)		
High	1.00 (0.95–1.04)		
**Conditional cash transfer recipient**	1.00 (0.96–1.04)		
**Facility type**			
Basic	1.00		
Complete	0.92 (0.86–0.99)		
**Travel time to delivery facility**			
<30 min.	1.00		
30 min. <1 hr.	1.00 (0.94–1.05)		
1 hr. to <2 hr.	0.96 (0.91–1.02)		
> 2 hr.	0.99 (0.94–1.04)		
**Caesarean section**	0.95 (0.91–1.00)		
**Staff spoke your language**	1.00 (0.96–1.05)		
**Allowed to be accompanied?**	1.00 (0.95–1.05)		
**Allowed to wear clothing of choice?**	1.00 (0.93–1.06)		
**Supplied bed allowing for position of choice?**	1.04 (0.99–1.08)		
**Allowed to consume beverage of choice**	1.01 (0.95–1.08)		
**Treated with respect**	1.29 (1.16–1.44)	2.70 (1.57–4.63)	1.41 (0.97–2.04)
**Allowed to select the birth position**	1.03 (0.99–1.08)		
**HEALTH FACILITY SURVEY**			
**Delivery room adaptation**	1.10 (1.02–1.19)		
**Staff speak an indigenous language**	1.09 (1.01–1.16)		1.10 (1.02–1.19)
**Allow accompaniment when coming for delivery**	1.06 (0.99–1.15)		
**Allow accompaniment by community health worker**	1.15 (1.09–1.22)	1.20 (1.10–1.32)	1.15 (1.05–1.26)
**Allow accompaniment by traditional birth attendant**	1.09 (1.01–1.17)		
**Allowable position: in a bed**	1.09 (1.02–1.16)		
**Allowable position: in a chair**	0.94 (0.86–1.04)		
**Allowable position: on knees**	0.97 (0.89–1.06)		
**Allowable position: sitting**	1.07 (0.99–1.15)		
**Allowable position: squatting**	0.97 (0.90–1.06)		
**Allowable position: standing**	0.98 (0.90–1.06)		
**Allowable position: vertically**	1.10 (1.03–1.18)		
**Country**	1.00		
Guatemala	1.10 (1.03–1.17)	1.00	1.00
Mexico	1.10 (1.02–1.19)	1.04 (0.94–1.16)	1.11 (1.00–1.23)

**Fig 1:**
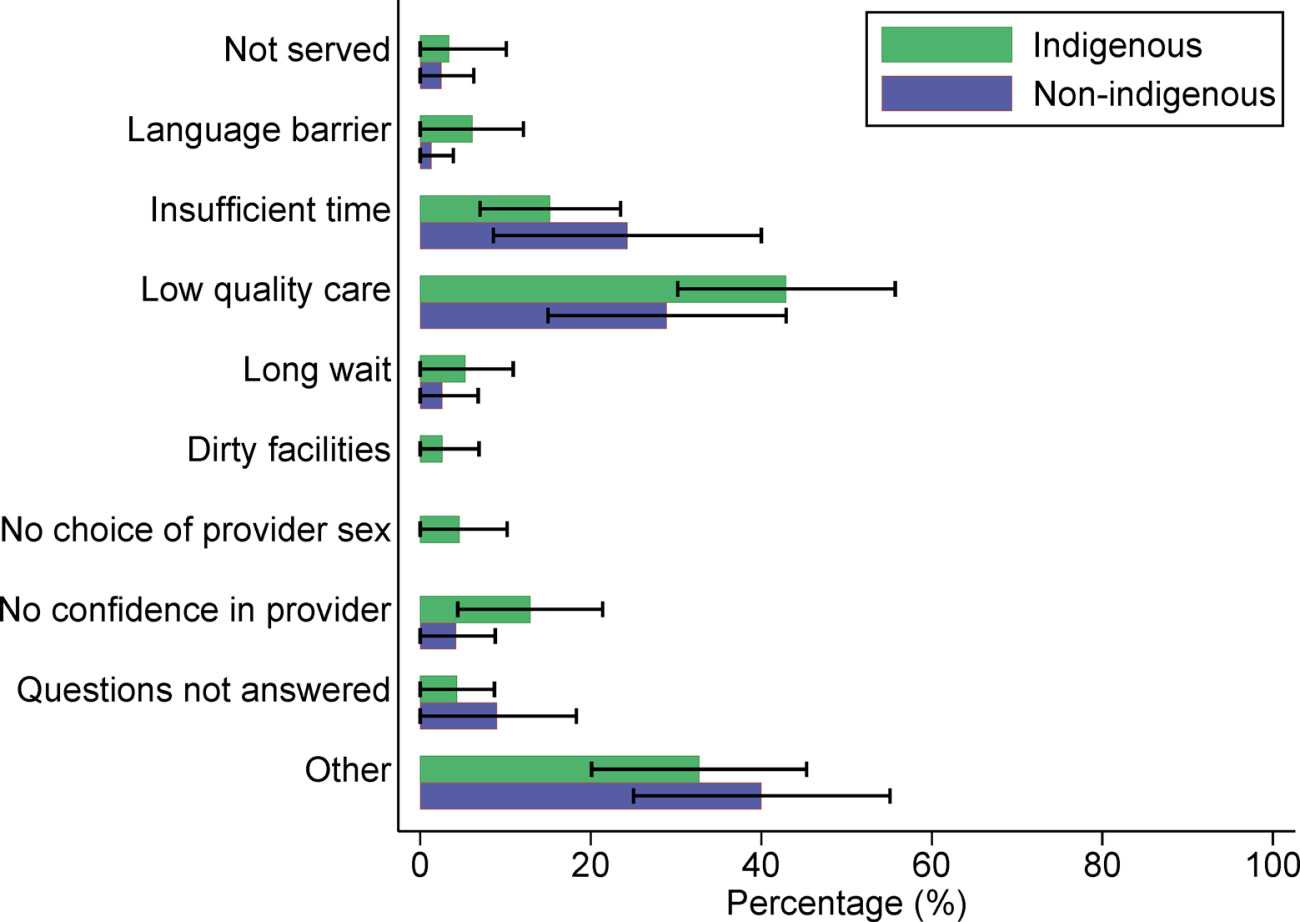
Reasons why Guatemalan women would not give birth in the same health facility again (n = 174).

**Fig 2:**
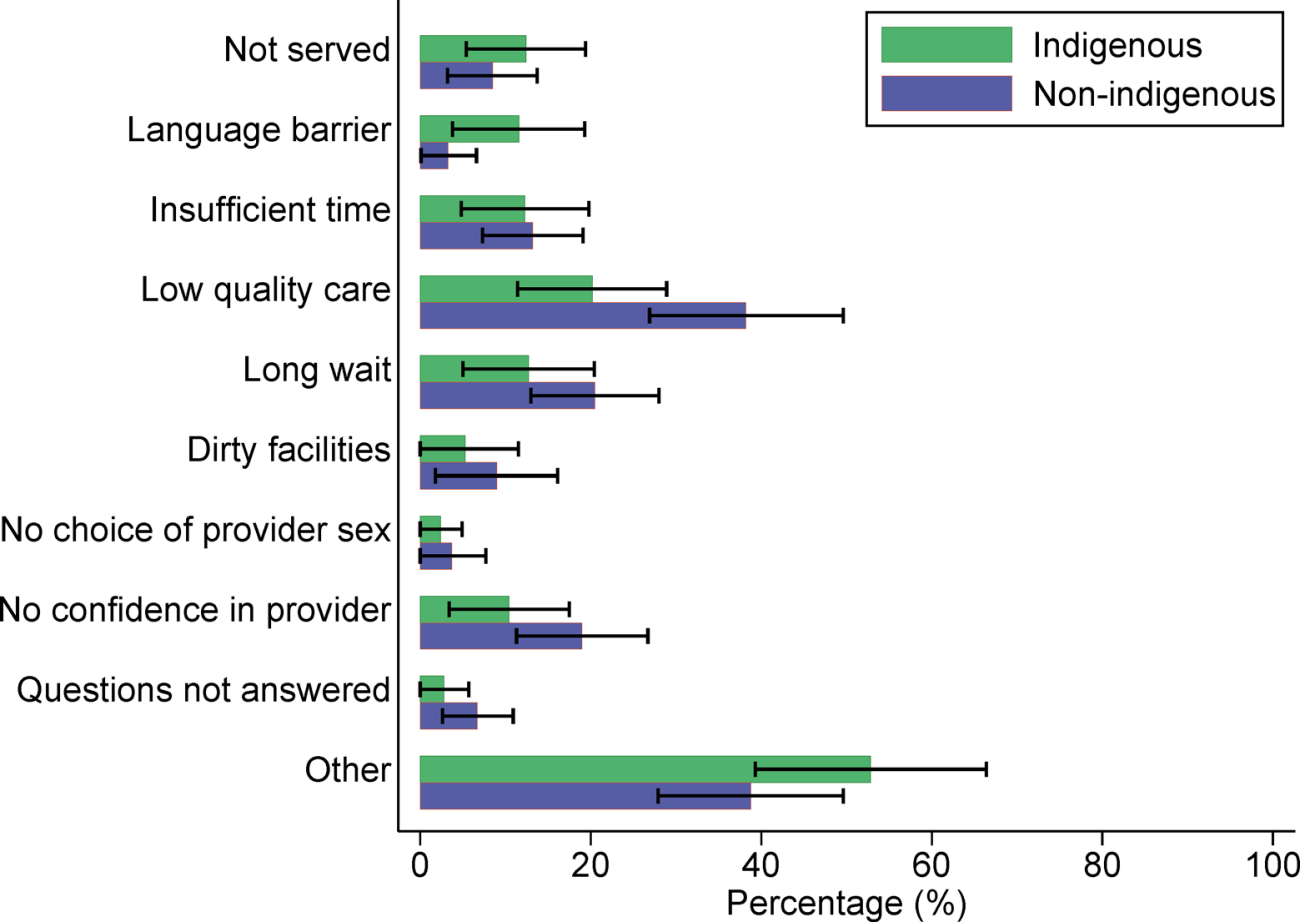
Reasons why Mexican women would not give birth in the same health facility again (n = 323).

**Fig 3:**
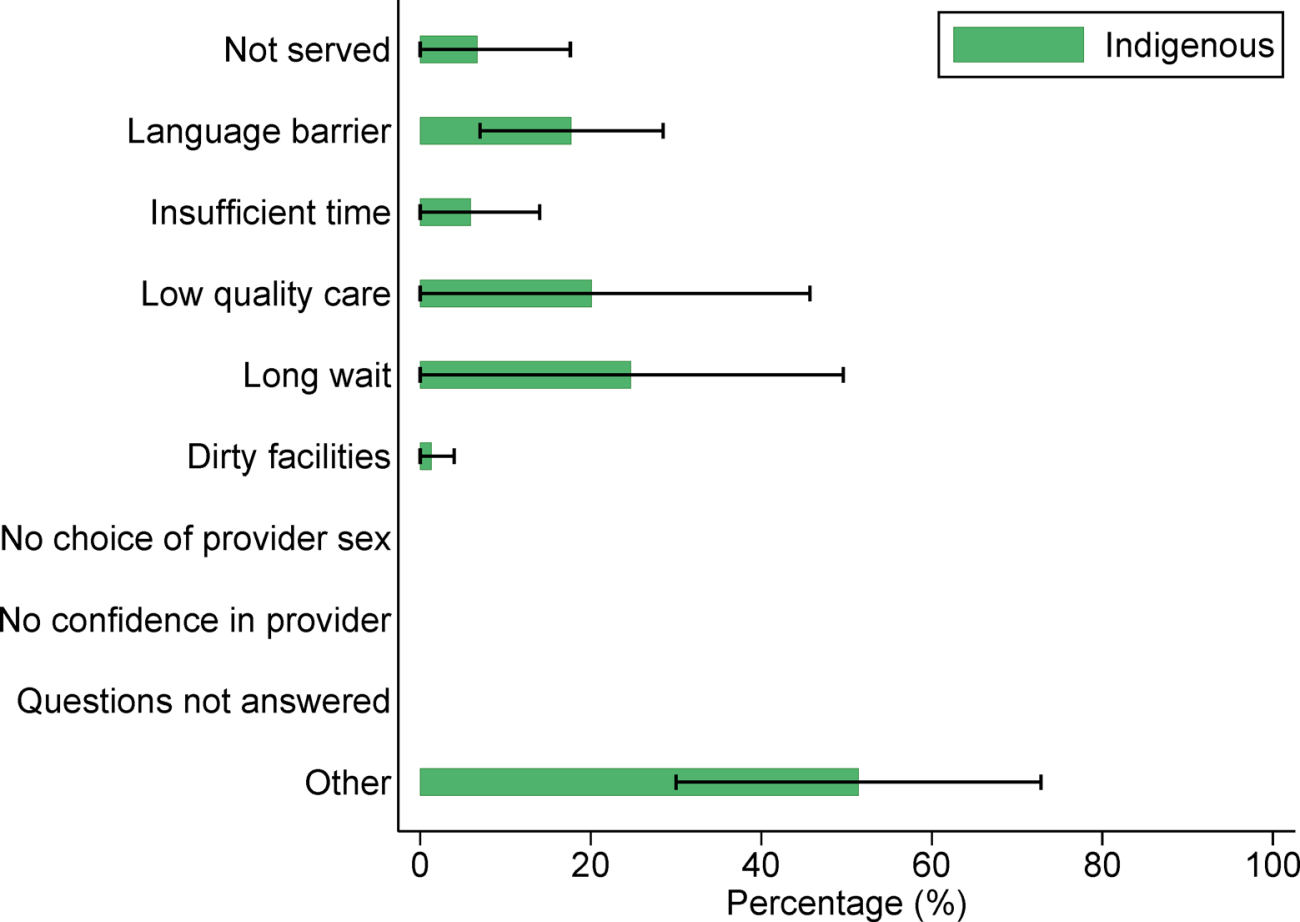
Reasons why Panamanian women would not give birth in the same health facility again (n = 51).

In our exploratory analyses of dissatisfaction among women who delivered in a basic or complete level facility, indigenous Guatemalan women (n = 99) most commonly cited perceived low quality of care as a reason they would not give birth in the same facility again. Among non-indigenous women (n = 75), the most common reason was not identified (“other”), followed by perceived low quality of care (Fig 1). Among the 323 Mexican women who were dissatisfied with their experience of delivering in a health facility, both indigenous and non-indigenous women most often cited “other” reasons, followed by perceived low quality of care (Fig 2). Of the 51 Panamanian women who were not satisfied with their institutional delivery, “other” reasons were most commonly cited, followed by long waiting times (Fig 3).

**Fig 4:**
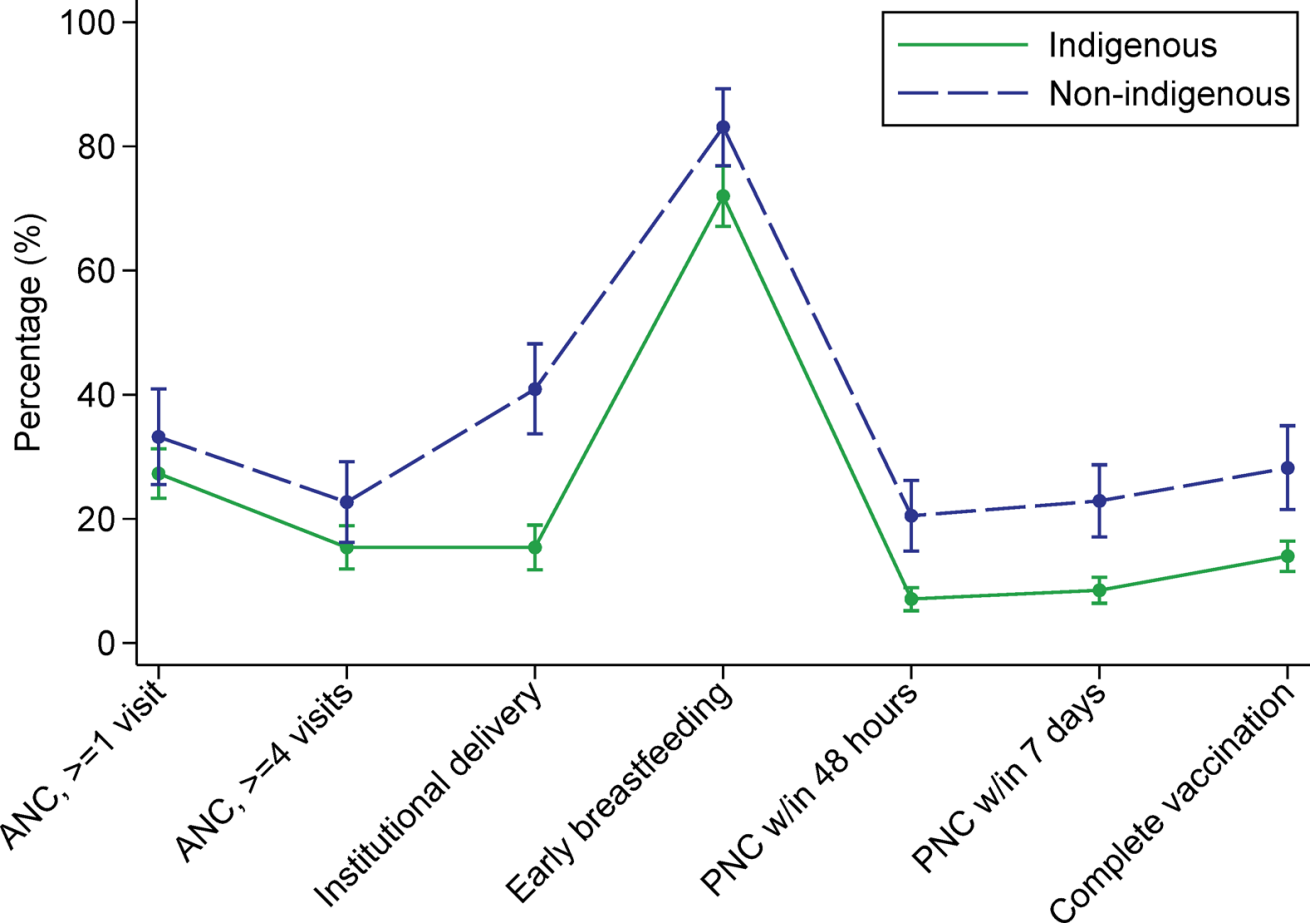
Guatemalan women’s utilization of services along the continuum of care. **Abbreviations: ANC, skilled antenatal care; PNC, post-natal care.

**Fig 5:**
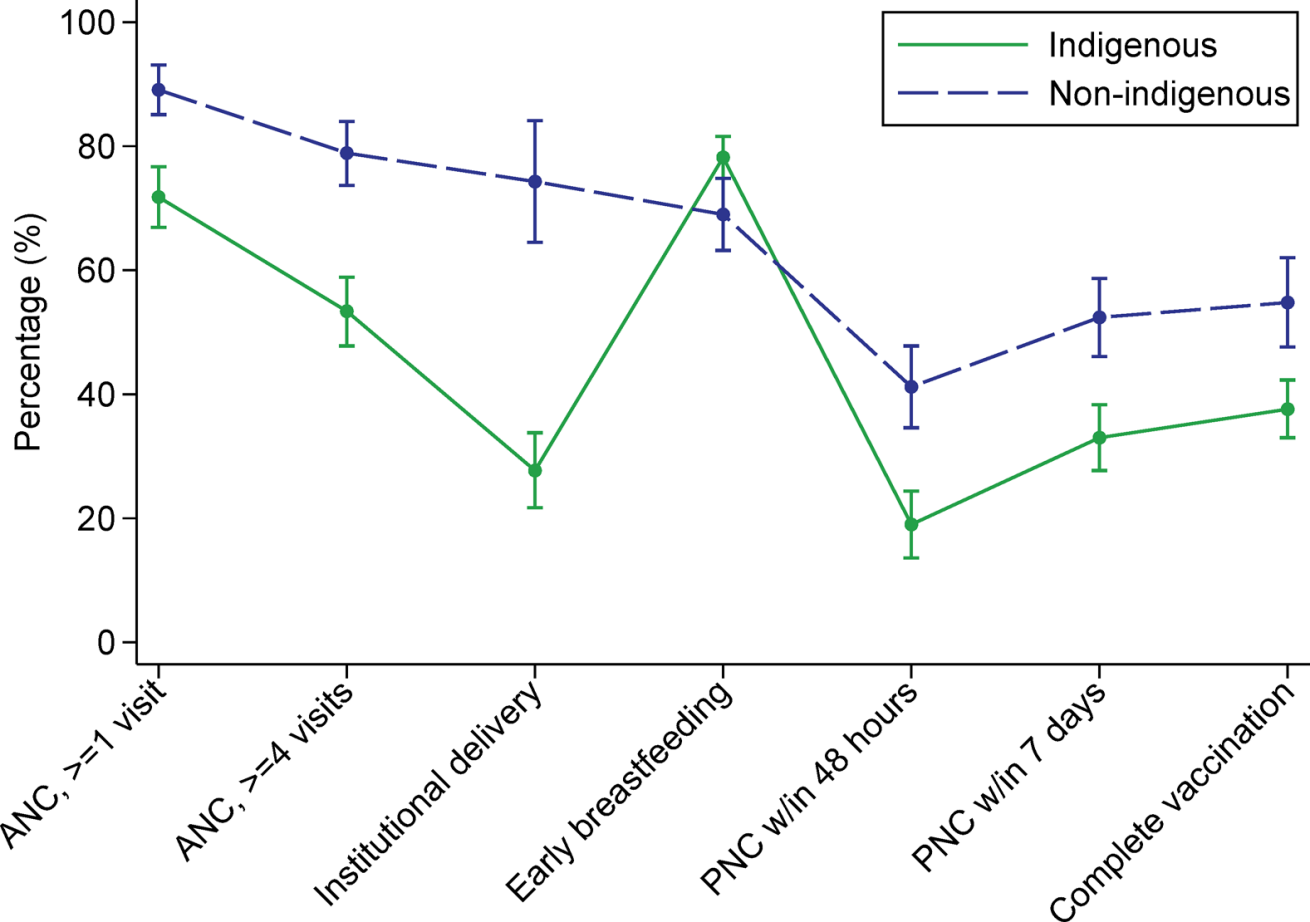
Mexican women’s utilization of services along the continuum of care. **Abbreviations: ANC, skilled antenatal care; PNC, post-natal care.

**Fig 6:**
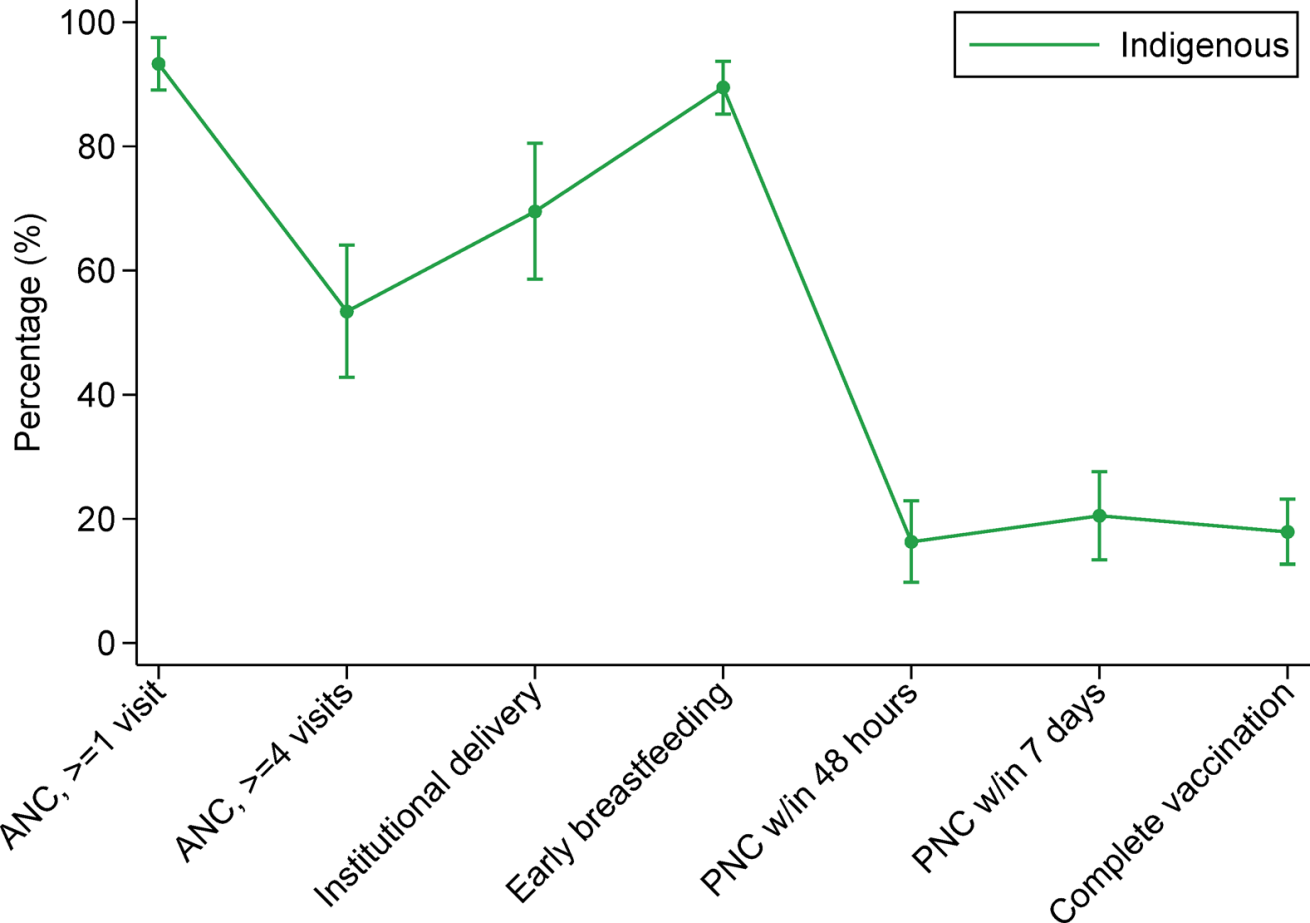
Panamanian women’s utilization of services along the continuum of care. **Abbreviations: ANC, skilled antenatal care; PNC, post-natal care.

The continuum of care graphs for indigenous and non-indigenous women in Guatemala were similar in shape, but indigenous women were less likely to have had an institutional delivery, PNC within 48 hours or 7 days, and a child who was completely vaccinated (Fig 4). In Mexico, indigenous women had a lower percentage of ANC (≥1 or ≥4 visits), institutional delivery, PNC (within 48 hours or 7 days) and complete vaccination (Fig 5). In Panama, there was no comparison group for the indigenous women, but the graph revealed a substantial drop in coverage in the post-natal period (Fig 6).

## Discussion

To our knowledge, this is the first multicountry analysis of correlates of institutional delivery and satisfaction among indigenous women. We found that, compared to non-indigenous women, indigenous women were substantially and significantly less likely to deliver in a health facility. We also found that approximately one out of six women in our study who had an institutional delivery would not return to that facility for a subsequent birth. The correlates of having an institutional delivery and satisfaction with that delivery differed somewhat between indigenous and non-indigenous women.

The strongest correlate of institutional delivery, for indigenous and non-indigenous women, was having one or more ANC visits. This is not unexpected and has been reported by others [9,12]. However, it is far from clear whether this is a causal relationship. Women who were inclined to pursue ANC may have done so as a precursor to having an institutional delivery. Interestingly, women participating in these CCT programs are incentivized to receive ANC, but institutional delivery is not a condition for payment, and women in these programs are less likely to have an institutional birth. Primiparity and education were positively associated with institutional delivery for indigenous women, which is in alignment with some previous studies [9,12]. That travel time to the closest facility was not generally associated with institutional delivery was surprising. Previous studies have shown that longer distances to health facilities are inversely related with utilization of those facilities [9], with the effect possibly being more pronounced among indigenous women [29]. We suspect that this might be because there was relatively little variation in travel time, with approximately 80% of women reporting times less than one hour. In addition, cultural factors, such as decision-making power resting with the husband or mother-in-law [13,17], are also known to be associated with institutional delivery. Unfortunately, we lacked data to assess the impact of family power dynamics in our study.

Our data show that many first-time indigenous mothers are willing to deliver in a health facility. It is therefore imperative that health systems focus attention on correlates of satisfaction in order to retain these women in the system and improve utilization of birthing facilities among higher parity women. Chief among the correlates of satisfaction is being treated with respect, which nearly tripled the likelihood of satisfaction for non-indigenous women overall and nearly doubled the likelihood of satisfaction among indigenous Guatemalan women. Subsequent qualitative studies should seek to better understand how these women perceive respect and what type of training health facility personnel need for improvement in this area. It is also apparent that both indigenous and non-indigenous women appreciate being given choices in the birthing process. Policy changes that ensure women are allowed to be accompanied by a community health worker and the freedom to select the birthing position and clothing of choice would be logical starting points. While acknowledging that much has already been done to improve the provision of culturally adapted choices across the region, more must be done in order to encourage women to return to the health facility for subsequent births.

This study has several limitations. First, ethnicity was solely defined by language, whereas ethnicity in the region is a complex social construct [30]. However, we followed the methods of the demographic and health surveys (DHS) that considers language in the determination of ethnicity [11]. Second, we did not survey all of the health facilities utilized by the women in our household surveys. In addition to the reduction in power, this may have introduced unmeasured biases into our analysis of supply-side correlates of satisfaction. For example, in Mexico we were only able to conduct surveys among facilities run by the Ministry of Health and not among other public facilities, such as those run by the Social Security program. Third, we lacked a comparison group of non-indigenous women in Panama, which hinders our ability to distinguish attributes of indigenous women vs. economically and socially marginalized women in that country. We therefore excluded Panama from our cross-country analyses in order to prevent biasing these estimates. Fourth, our quantitative survey would have benefitted from a complementary qualitative study [31]. This would have prevented the need to dichotomize complex factors such as desiring a pregnancy. Fifth, the question which defined satisfaction assumed that the women had a viable choice among birthing facilities, which may not have been the case. In addition, as mentioned above, we lacked the ability to assess the impact of familial decision-making processes on utilization of birthing facilities. However, our study also has strengths, including its large sample size, representative population sampling, and use of standardized survey methodologies across countries and time, allowing for comparability.

In conclusion, we found low proportions of institutional delivery among the poorest quintile of women living in the region, with substantially lower proportions among Guatemalan and Mexican indigenous women. The health system must attempt to increase the use of birthing facilities by poor and indigenous women, possibly through increasing access to ANC and higher educational opportunities. The health systems should also continue to ensure the provision of culturally adapted services, with health facility personnel demonstrating respect toward women who have an institutional delivery, in order to improve their rates of return.

## Supporting Information

S1 Table Characteristics of indigenous and non-indigenous Guatemalan and Mexican women in the Salud Mesoamérica Initiative, 2011–2013. (DOCX)

S2 Table Correlates of institutional delivery among Guatemalan women in the Salud Mesoamérica Initiative, 2011–2013. (DOCX)

S3 Table Correlates of institutional delivery among Mexican women in the Salud Mesoamérica Initiative, 2011–2013. (DOCX)

S4 Table Correlates of institutional delivery among Panamanian women in the Salud Mesoamérica Initiative, 2011–2013. (DOCX)

S5 Table Correlates of satisfaction among women who gave birth in a health facility in Guatemala in the Salud Mesoamérica Initiative, 2011–2013. (DOCX)

S6 Table Correlates of satisfaction among women who gave birth in a health facility in Mexico in the Salud Mesoamérica Initiative, 2011–2013. (DOCX)

S7 Table Correlates of satisfaction among women who gave birth in a health facility in Panama in the Salud Mesoamérica Initiative, 2011–2013. (DOCX)
