# Supplementary material for: Institutional Delivery and Satisfaction among Indigenous and Poor Women in Guatemala, Mexico, and Panama
Source: PLoS One. 2016 Apr 27;11(4):e0154388. doi: 10.1371/journal.pone.0154388 (PMC4847770; doi:10.1371/journal.pone.0154388)
Supplement: S1 Table — (DOCX) [file pone.0154388.s001.docx]

**S1 Table.** Characteristics of indigenous and non-indigenous Guatemalan and Mexican women in the Salud Mesoamérica Initiative, 2011-2013.

|  | **Non-indigenous**  **n=2,727** | **Indigenous**  **n=7,425** | **P** |
| --- | --- | --- | --- |
| **WOMEN** |  |  |  |
| **Age (years)** |  |  | <0.001 |
| 15-24 | 36.9 (34.0-40.0) | 35.3 (32.9-37.7) |  |
| 25-34 | 47.9 (44.6-51.2) | 43.0 (40.8-45.2) |  |
| 35-49 | 15.2 (13.2-17.5) | 21.8 (19.7-24.0) |  |
| **Education** |  |  | <0.001 |
| None | 7.4 (5.9-9.2) | 23.9 (20.9-27.2) |  |
| Primary | 45.6 (39.7-51.7) | 55.8 (52.3-59.2) |  |
| Secondary or higher | 47.0 (41.0-53.2) | 20.3 (17.6-23.4) |  |
| **Literate** | 90.6 (88.0-92.6) | 69.5 (66.2-72.5) | <0.001 |
| **Wealth index** |  |  | <0.001 |
| Low | 40.9 (35.0-47.1) | 66.9 (62.8-70.7) |  |
| Medium | 33.3 (29.9-36.8) | 23.8 (21.0-26.9) |  |
| High | 25.8 (21.8-30.3) | 9.3 (7.7-11.2) |  |
| **Conditional cash transfer recipient** | 48.3 (41.2-55.4) | 71.9 (68.6-74.9) | <0.001 |
| **Married** | 90.9 (89.1-92.3) | 94.1 (93.2-94.9) | <0.001 |
| **Wanted the pregnancy** | 75.8 (72.4-79.0) | 83.3 (80.9-85.4) | <0.001 |
| **Primiparous** | 24.7 (21.9-27.8) | 13.6 (12.3-14.9) | <0.001 |
| **Traditional birth attendant assisted in delivery** | 2.0 (0.9-4.6) | 2.5 (1.1-5.6) | 0.687 |
| **Woman gave birth in a facility** | 71.3 (63.4-78.2) | 27.6 (23.0-32.8) | <0.001 |
| **Caesarean section*** | 35.4 (31.4-39.7) | 25.9 (23.1-28.9) | <0.001 |
| **Emergency C-section*** | 69.6 (63.5-75.1) | 79.4 (73.6-84.1) | 0.019 |
| **Delivery travel time*** |  |  | <0.001 |
| <30 min. | 36.6 (30.2-43.4) | 18.0 (13.6-23.4) |  |
| 30 min. <1 hr. | 23.4 (19.1-28.3) | 22.6 (17.0-29.3) |  |
| 1 hr. to <2 hr. | 15.2 (11.0-20.6) | 21.9 (17.0-27.8) |  |
| > 2 hr. | 24.9 (19.7-30.8) | 37.5 (27.9-48.3) |  |
| **CLOSET HEALTH FACILITY** |  |  |  |
| **Facility type** |  |  | 0.863 |
| ambulatory | 42.2 (29.7-55.9) | 40.3 (30.1-51.4) |  |
| basic | 39.7 (25.7-55.7) | 43.5 (32.5-55.1) |  |
| complete | 18.0 (10.8-28.6) | 16.3 (10.6-24.1) |  |
| **Delivery room adapted to indigenous populations†** | 13.4 (5.0-31.0) | 19.6 (9.1-37.3) | 0.419 |
| **Facility adapts services to the socio-cultural condition of the women** | 54.8 (35.9-72.4) | 72.3 (56.7-83.9) | 0.113 |
| **Medical staff speak an indigenous language** | 35.2 (20.6-53.2) | 66.0 (50.6-78.6) | 0.003 |
| **Allow accompaniment when coming for delivery†** | 34.6 (20.4-52.1) | 54.3 (38.5-69.3) | 0.055 |
| **Allow accompaniment by community health worker†** | 32.4 (15.2-56.2) | 7.4 (2.8-18.3) | 0.007 |
| **Allow accompaniment by traditional birth attendant†** | 99.9 (99.3-100.0) | 81.1 (52.1-94.4) | <0.001 |
| **Allowable position: in a bed†** | 27.1 (15.5-43.0) | 38.6 (25.9-53.2) | 0.190 |
| **Allowable position: in a chair†** | 6.3 (2.8-13.5) | 3.7 (1.8-7.6) | 0.301 |
| **Allowable position: on knees†** | 9.7 (5.1-17.7) | 15.0 (9.7-22.5) | 0.200 |
| **Allowable position: sitting†** | 8.4 (4.2-16.1) | 10.1 (6.2-16.1) | 0.609 |
| **Allowable position: squatting†** | 8.4 (4.2-16.1) | 10.4 (6.5-16.4) | 0.567 |
| **Allowable position: standing†** | 8.6 (4.3-16.3) | 11.2 (6.9-17.5) | 0.462 |
| **Allowable position: vertically†** | 28.7 (14.3-49.2) | 32.0 (18.9-48.8) | 0.731 |

*Asked only of women who gave birth in a facility

† Applicable only to women whose closest birth facility had the capacity to attend births, i.e., those that were type basic or complete.
