# Supplementary material for: Institutional Delivery and Satisfaction among Indigenous and Poor Women in Guatemala, Mexico, and Panama
Source: PLoS One. 2016 Apr 27;11(4):e0154388. doi: 10.1371/journal.pone.0154388 (PMC4847770; doi:10.1371/journal.pone.0154388)
Supplement: S2 Table — (DOCX) [file pone.0154388.s002.docx]

**S2 Table.** Correlates of institutional delivery among Guatemalan women in the Salud Mesoamérica Initiative, 2011-2013.

|  | **Univariate** |  | **Non-indigenous Multivariate** |  | **Indigenous Multivariate** |
| --- | --- | --- | --- | --- | --- |
|  | **n=4,454** |  | **n=655** |  | **n=3,226** |
|  | **RR (95% CI)** |  | **aRR (95% CI)** |  | **aRR (95% CI)** |
| **Age (years)** |  |  |  |  |  |
| 15-24 | 1.00 |  |  |  |  |
| 25-34 | 0.84 (0.72-0.99) |  |  |  |  |
| 35-49 | 0.65 (0.52-0.82) |  |  |  |  |
| **Education** |  |  |  |  |  |
| None | 1.00 |  | 1.00 |  | 1.00 |
| Primary | 2.20 (1.72-2.82) |  | 1.15 (0.74-1.78) |  | 1.61 (1.20-2.16) |
| Secondary or higher | 5.37 (3.97-7.28) |  | 1.86 (1.20-2.88) |  | 2.59 (1.83-3.67) |
| **Literate** | 2.62 (2.03-3.37) |  |  |  |  |
| **Indigenous ethnicity** | 0.37 (0.29-0.46) |  |  |  |  |
| **Married** | 0.72 (0.59-0.88) |  |  |  |  |
| **Urban residence** | 2.29 (1.59-3.28) |  |  |  | 1.58 (1.18-2.12) |
| **Wealth index** |  |  |  |  |  |
| Low | 1.00 |  |  |  |  |
| Medium | 1.70 (1.40-2.07) |  |  |  |  |
| High | 2.50 (2.01-3.12) |  |  |  |  |
| **Conditional cash transfer recipient** | 0.49 (0.39-0.60) |  |  |  | 0.68 (0.53-0.88) |
| **Wanted the pregnancy** | 0.77 (0.60-0.97) |  |  |  |  |
| **Primiparous** | 1.94 (1.67-2.24) |  |  |  | 1.26 (1.03-1.54) |
| **≥1 skilled antenatal care visit** | 3.25 (2.67-3.96) |  | 1.49 (1.20-1.84) |  | 2.11 (1.62-2.76) |
| **≥4 skilled antenatal care visits** | 3.12 (2.55-3.82) |  |  |  |  |
| **Advised to give birth in a health facility** | 2.76 (2.34-3.26) |  | 1.24 (0.99-1.55) |  | 1.53 (1.17-2.00) |
| **Advised to create a transportation plan** | 1.99 (1.63-2.43) |  |  |  |  |
| **Informed that should have a  c-section** | 3.30 (2.76-3.95) |  | 1.33 (1.07-1.66) |  | 1.83 (1.47-2.28) |
| **Closest health facility type** |  |  |  |  |  |
| Ambulatory | 1.00 |  | 1.00 |  |  |
| Basic | 1.58 (1.03-2.44) |  | 1.33 (0.97-1.81) |  |  |
| Complete | 2.17 (0.90-5.21) |  | 1.80 (1.16-2.79) |  |  |
| **Travel time to closest delivery facility** |  |  |  |  |  |
| <30 min. | 1.00 |  |  |  |  |
| 30 min. <1 hr. | 0.67 (0.48-0.92) |  |  |  |  |
| 1 hr. to <2 hr. | 0.65 (0.44-0.97) |  |  |  |  |
| > 2 hr. | 0.53 (0.34-0.80) |  |  |  |  |
