# Supplementary material for: Institutional Delivery and Satisfaction among Indigenous and Poor Women in Guatemala, Mexico, and Panama
Source: PLoS One. 2016 Apr 27;11(4):e0154388. doi: 10.1371/journal.pone.0154388 (PMC4847770; doi:10.1371/journal.pone.0154388)
Supplement: S3 Table — (DOCX) [file pone.0154388.s003.docx]

**S3 Table.** Correlates of institutional delivery among Mexican women in the Salud Mesoamérica Initiative, 2011-2013.

|  | **Univariate** |  | **Non-indigenous Multivariate** |  | **Indigenous Multivariate** |
| --- | --- | --- | --- | --- | --- |
|  | **n=5,698** |  | **n=1,594** |  | **n=3,952** |
|  | **RR (95% CI)** |  | **aRR (95% CI)** |  | **aRR (95% CI)** |
| **Age (years)** |  |  |  |  |  |
| 15-24 | 1.00 |  |  |  |  |
| 25-34 | 0.94 (0.85-1.04) |  |  |  |  |
| 35-49 | 0.87 (0.75-1.01) |  |  |  |  |
| **Education** |  |  |  |  |  |
| None | 1.00 |  | 1.00 |  | 1.00 |
| Primary | 1.39 (1.14-1.69) |  | 0.95 (0.76-1.19) |  | 1.03 (0.80-1.32) |
| Secondary or higher | 2.67 (2.17-3.28) |  | 1.04 (0.83-1.29) |  | 1.43 (1.16-1.78) |
| **Literate** | 1.99 (1.67-2.37) |  | 1.24 (0.98-1.58) |  |  |
| **Indigenous ethnicity** | 0.39 (0.32-0.48) |  |  |  |  |
| **Married** | 0.77 (0.66-0.89) |  |  |  | 0.76 (0.61-0.96) |
| **Urban residence** | 2.08 (1.63-2.64) |  | 1.18 (0.95-1.46) |  |  |
| **Wealth index** |  |  |  |  |  |
| Low | 1.00 |  | 1.00 |  |  |
| Medium | 1.44 (1.23-1.69) |  | 1.02 (0.91-1.15) |  |  |
| High | 1.98 (1.67-2.35) |  | 1.13 (1.01-1.27) |  |  |
| **Conditional cash transfer recipient** | 0.52 (0.46-0.60) |  | 0.81 (0.72-0.92) |  | 0.65 (0.55-0.78) |
| **Wanted the pregnancy** | 0.76 (0.68-0.85) |  |  |  | 0.80 (0.70-0.91) |
| **Primiparous** | 1.70 (1.51-1.91) |  |  |  | 1.48 (1.26-1.74) |
| **≥1 skilled antenatal care visit** | 3.19 (2.47-4.11) |  | 1.51 (1.15-1.99) |  | 1.94 (1.38-2.72) |
| **≥4 skilled antenatal care visits** | 2.42 (2.02-2.90) |  |  |  | 1.42 (1.17-1.72) |
| **Advised to give birth in a health facility** | 2.25 (1.95-2.59) |  |  |  | 1.46 (1.17-1.83) |
| **Advised to create a transportation plan** | 1.68 (1.44-1.98) |  |  |  |  |
| **Informed that should have a  c-section** | 2.04 (1.80-2.32) |  | 1.10 (1.01-1.20) |  | 1.40 (1.20-1.64) |
| **Closest health facility type** |  |  |  |  |  |
| Ambulatory | 1.00 |  |  |  |  |
| Basic | 0.87 (0.61-1.25) |  |  |  |  |
| Complete | 0.85 (0.58-1.26) |  |  |  |  |
| **Travel time to closest delivery facility** |  |  |  |  |  |
| <30 min. | 1.00 |  |  |  |  |
| 30 min. <1 hr. | 0.89 (0.71-1.12) |  |  |  |  |
| 1 hr. to <2 hr. | 0.89 (0.67-1.18) |  |  |  |  |
| > 2 hr. | 0.95 (0.58-1.57) |  |  |  |  |
