# Supplementary material for: Institutional Delivery and Satisfaction among Indigenous and Poor Women in Guatemala, Mexico, and Panama
Source: PLoS One. 2016 Apr 27;11(4):e0154388. doi: 10.1371/journal.pone.0154388 (PMC4847770; doi:10.1371/journal.pone.0154388)
Supplement: S4 Table — (DOCX) [file pone.0154388.s004.docx]

**S4 Table.** Correlates of institutional delivery among Panamanian women in the Salud Mesoamérica Initiative, 2011-2013.

|  | **Univariate** |  | **Indigenous Multivariate** |
| --- | --- | --- | --- |
|  | **n=743** |  | **n=695** |
|  | **RR (95% CI)** |  | **aRR (95% CI)** |
| **Age (years)** |  |  |  |
| 15-24 | 1.00 |  |  |
| 25-34 | 1.07 (0.92-1.25) |  |  |
| 35-49 | 1.09 (0.97-1.23) |  |  |
| **Education** |  |  |  |
| None | 1.00 |  |  |
| Primary | 0.92 (0.78-1.08) |  |  |
| Secondary or higher | 1.11 (0.92-1.33) |  |  |
| **Literate** | 1.00 (0.83-1.22) |  |  |
| **Married** | 0.87 (0.75-1.01) |  |  |
| **Wealth index** |  |  |  |
| Low | 1.00 |  |  |
| Medium | 1.07 (0.89-1.29) |  |  |
| High | 1.04 (0.84-1.29) |  |  |
| **Conditional cash transfer recipient** | 0.88 (0.76-1.02) |  |  |
| **Wanted the pregnancy** | 1.03 (0.87-1.22) |  |  |
| **Primiparous** | 1.16 (1.02-1.33) |  | 1.15 (1.01-1.30) |
| **≥1 skilled antenatal care visit** | 1.45 (1.02-2.07) |  | 1.35 (0.92-1.99) |
| **≥4 skilled antenatal care visits** | 1.05 (0.90-1.21) |  |  |
| **Advised to give birth in a health facility** | 0.99 (0.87-1.13) |  |  |
| **Advised to create a transportation plan** | 1.37 (1.18-1.59) |  | 1.34 (1.17-1.54) |
| **Informed that should have a c-section** | 1.22 (1.04-1.44) |  |  |
| **Closest health facility type** |  |  |  |
| Ambulatory | 1.00 |  |  |
| Basic | 1.06 (0.73-1.54) |  |  |
| **Travel time to closest delivery facility** |  |  |  |
| <30 min. | 1.00 |  |  |
| 30 min. <1 hr. | 1.02 (0.77-1.36) |  |  |
| 1 hr. to <2 hr. | 1.09 (0.82-1.44) |  |  |
| > 2 hr. | 0.79 (0.59-1.07) |  |  |
