# Supplementary material for: Institutional Delivery and Satisfaction among Indigenous and Poor Women in Guatemala, Mexico, and Panama
Source: PLoS One. 2016 Apr 27;11(4):e0154388. doi: 10.1371/journal.pone.0154388 (PMC4847770; doi:10.1371/journal.pone.0154388)
Supplement: S5 Table — (DOCX) [file pone.0154388.s005.docx]

**S5 Table.** Correlates of satisfaction among women who gave birth in a health facility in Guatemala in the Salud Mesoamérica Initiative, 2011-2013.

|  | **Univariate** |  | **Non-indigenous Multivariate** |  | **Indigenous Multivariate** |
| --- | --- | --- | --- | --- | --- |
|  | **n=516** |  | **n=232** |  | **n=224** |
|  | **RR (95% CI)** |  | **aRR (95% CI)** |  | **aRR (95% CI)** |
| **HOUSEHOLD SURVEY DATA** |  |  |  |  |  |
| **Age (years)** |  |  |  |  |  |
| 15-24 | 1.00 |  |  |  |  |
| 25-34 | 1.01 (0.91-1.11) |  |  |  |  |
| 35-49 | 0.90 (0.78-1.03) |  |  |  |  |
| **Education** |  |  |  |  |  |
| None | 1.00 |  |  |  |  |
| Primary | 0.94 (0.84-1.07) |  |  |  |  |
| Secondary or higher | 0.95 (0.82-1.10) |  |  |  |  |
| **Literate** | 0.97 (0.86-1.11) |  |  |  |  |
| **Indigenous ethnicity** | 0.96 (0.86-1.07) |  |  |  |  |
| **Married** | 0.97 (0.87-1.09) |  |  |  |  |
| **Urban residence** | 1.08 (0.96-1.21) |  |  |  |  |
| **Wealth index** |  |  |  |  |  |
| Low | 1.00 |  |  |  |  |
| Medium | 1.04 (0.93-1.16) |  |  |  |  |
| High | 1.04 (0.92-1.19) |  |  |  |  |
| **Conditional cash transfer recipient** | 0.93 (0.82-1.07) |  |  |  |  |
| **Facility type** |  |  |  |  |  |
| Basic | 1.00 |  |  |  |  |
| Complete | 0.88 (0.78-1.00) |  |  |  |  |
| **Travel time to delivery facility** |  |  |  |  |  |
| <30 min. | 1.00 |  |  |  |  |
| 30 min. <1 hr. | 0.99 (0.89-1.10) |  |  |  |  |
| 1 hr. to <2 hr. | 0.81 (0.68-0.96) |  |  |  |  |
| > 2 hr. | 0.89 (0.79-1.00) |  |  |  |  |
| **Caesarean section** | 0.90 (0.81-1.01) |  |  |  |  |
| **Staff spoke your language** | 1.16 (1.03-1.29) |  |  |  | 1.17 (1.05-1.31) |
| **Allowed to be accompanied?** | 1.09 (0.99-1.20) |  |  |  |  |
| **Allowed to wear clothing of choice?** | 1.17 (1.07-1.27) |  |  |  |  |
| **Supplied bed allowing for position of choice?** | 1.20 (1.10-1.30) |  | 1.21 (1.03-1.42) |  |  |
| **Allowed to consume beverage of choice** | 1.15 (1.06-1.26) |  |  |  |  |
| **Treated with respect** | 1.58 (1.17-2.13) |  |  |  | 1.88 (1.08-3.25) |
| **Allowed to select the birth position** | 1.15 (1.06-1.25) |  |  |  | 1.16 (1.02-1.32) |
| **HEALTH FACILITY SURVEY DATA** |  |  |  |  |  |
| **Delivery room adaptation** | 1.14 (1.01-1.29) |  |  |  |  |
| **Staff speak an indigenous language** | 0.92 (0.79-1.07) |  |  |  |  |
| **Allow accompaniment when coming for delivery** | 1.14 (1.00-1.29) |  |  |  |  |
| **Allow accompaniment by community health worker** | 1.21 (1.11-1.32) |  | 1.11 (1.03-1.20) |  | 1.10 (1.00-1.21) |
| **Allow accompaniment by traditional birth attendant** | 1.13 (1.00-1.28) |  |  |  |  |
| **Allowable position: in a bed** | 1.19 (1.05-1.35) |  | 1.26 (1.04-1.53) |  |  |
| **Allowable position: in a chair** | 0.87 (0.79-0.96) |  |  |  |  |
| **Allowable position: on knees** | 0.93 (0.83-1.05) |  |  |  |  |
| **Allowable position: sitting** | 1.12 (0.99-1.27) |  |  |  |  |
| **Allowable position: squatting** | 0.94 (0.83-1.06) |  |  |  |  |
| **Allowable position: standing** | 0.94 (0.83-1.06) |  |  |  |  |
| **Allowable position: vertically** | 1.00 (0.86-1.16) |  |  |  |  |
