# Supplementary material for: Institutional Delivery and Satisfaction among Indigenous and Poor Women in Guatemala, Mexico, and Panama
Source: PLoS One. 2016 Apr 27;11(4):e0154388. doi: 10.1371/journal.pone.0154388 (PMC4847770; doi:10.1371/journal.pone.0154388)
Supplement: S6 Table — (DOCX) [file pone.0154388.s006.docx]

**S6 Table.** Correlates of satisfaction among women who gave birth in a health facility in Mexico in the Salud Mesoamérica Initiative, 2011-2013.

|  | **Univariate** |  | **Non-indigenous Multivariate** |  | **Indigenous Multivariate** |
| --- | --- | --- | --- | --- | --- |
|  | **n=698** |  | **n=295** |  | **n=369** |
|  | **RR (95% CI)** |  | **aRR (95% CI)** |  | **aRR (95% CI)** |
| **HOUSEHOLD SURVEY DATA** |  |  |  |  |  |
| **Age (years)** |  |  |  |  |  |
| 15-24 | 1.00 |  |  |  |  |
| 25-34 | 0.95 (0.91-1.01) |  |  |  |  |
| 35-49 | 0.97 (0.91-1.03) |  |  |  |  |
| **Education** |  |  |  |  |  |
| None | 1.00 |  | 1.00 |  | 1.00 |
| Primary | 0.97 (0.91-1.03) |  | 0.90 (0.83-0.98) |  | 0.93 (0.86-1.00) |
| Secondary or higher | 0.94 (0.88-1.00) |  | 0.89 (0.82-0.96) |  | 0.87 (0.77-0.98) |
| **Literate** | 0.96 (0.90-1.02) |  |  |  |  |
| **Indigenous ethnicity** | 1.02 (0.98-1.06) |  |  |  |  |
| **Married** | 1.05 (0.97-1.14) |  |  |  |  |
| **Urban residence** | 0.99 (0.95-1.03) |  |  |  |  |
| **Wealth index** |  |  |  |  |  |
| Low | 1.00 |  |  |  |  |
| Medium | 0.96 (0.91-1.01) |  |  |  |  |
| High | 1.00 (0.95-1.05) |  |  |  |  |
| **Conditional cash transfer recipient** | 0.99 (0.95-1.04) |  |  |  |  |
| **Facility type** |  |  |  |  |  |
| Basic | 1.00 |  |  |  |  |
| Complete | 0.92 (0.85-1.00) |  |  |  |  |
| **Travel time to delivery facility** |  |  |  |  |  |
| <30 min. | 1.00 |  |  |  |  |
| 30 min. <1 hr. | 1.00 (0.94-1.06) |  |  |  |  |
| 1 hr. to <2 hr. | 0.97 (0.91-1.03) |  |  |  |  |
| > 2 hr. | 0.99 (0.94-1.05) |  |  |  |  |
| **Caesarean section** | 0.96 (0.91-1.00) |  |  |  |  |
| **Staff spoke your language** | 0.99 (0.95-1.04) |  |  |  |  |
| **Allowed to be accompanied?** | 1.00 (0.95-1.05) |  |  |  |  |
| **Allowed to wear clothing of choice?** | 0.99 (0.92-1.06) |  |  |  |  |
| **Supplied bed allowing for position of choice?** | 1.03 (0.99-1.08) |  |  |  |  |
| **Allowed to consume beverage of choice** | 1.00 (0.94-1.08) |  |  |  |  |
| **Treated with respect** | 1.28 (1.14-1.43) |  | 1.31 (1.13-1.52) |  | 1.36 (0.91-2.04) |
| **Allowed to select the birth position** | 1.02 (0.98-1.07) |  |  |  |  |
| **HEALTH FACILITY SURVEY DATA** |  |  |  |  |  |
| **Delivery room adaptation** | 1.10 (1.01-1.19) |  |  |  |  |
| **Staff speak an indigenous language** | 1.11 (1.03-1.19) |  |  |  | 1.10 (1.02-1.19) |
| **Allow accompaniment when coming for delivery** | 1.06 (0.97-1.17) |  |  |  |  |
| **Allow accompaniment by community health worker** | 1.00 |  |  |  |  |
| **Allow accompaniment by traditional birth attendant** | 1.10 (1.01-1.21) |  |  |  |  |
| **Allowable position: in a bed** | 1.08 (1.01-1.16) |  |  |  |  |
| **Allowable position: in a chair** | 1.18 (1.13-1.23) |  |  |  |  |
| **Allowable position: on knees** | 1.18 (1.13-1.23) |  |  |  |  |
| **Allowable position: sitting** | 1.18 (1.13-1.23) |  |  |  | 1.06 (0.98-1.13) |
| **Allowable position: squatting** | 1.18 (1.13-1.23) |  |  |  |  |
| **Allowable position: standing** | 1.18 (1.13-1.23) |  |  |  |  |
| **Allowable position: vertically** | 1.11 (1.03-1.20) |  |  |  |  |
