# Supplementary material for: Institutional Delivery and Satisfaction among Indigenous and Poor Women in Guatemala, Mexico, and Panama
Source: PLoS One. 2016 Apr 27;11(4):e0154388. doi: 10.1371/journal.pone.0154388 (PMC4847770; doi:10.1371/journal.pone.0154388)
Supplement: S7 Table — (DOCX) [file pone.0154388.s007.docx]

**S7 Table.** Correlates of satisfaction among women who gave birth in a health facility in Panama in the Salud Mesoamérica Initiative, 2011-2013.

|  | **Univariate** |  | **Indigenous Multivariate** |
| --- | --- | --- | --- |
|  | **n=356** |  | **n=318** |
|  | **RR (95% CI)** |  | **aRR (95% CI)** |
| **HOUSEHOLD SURVEY DATA** |  |  |  |
| **Age (years)** |  |  |  |
| 15-24 | 1.00 |  |  |
| 25-34 | 0.99 (0.89-1.11) |  |  |
| 35-49 | 0.93 (0.79-1.09) |  |  |
| **Education** |  |  |  |
| None | 1.00 |  |  |
| Primary | 1.04 (0.85-1.26) |  |  |
| Secondary or higher | 1.08 (0.89-1.31) |  |  |
| **Literate** | 1.06 (0.89-1.27) |  |  |
| **Married** | 1.01 (0.87-1.18) |  |  |
| **Wealth index** |  |  |  |
| Low | 1.00 |  |  |
| Medium | 0.97 (0.84-1.13) |  |  |
| High | 1.06 (0.93-1.20) |  |  |
| **Conditional cash transfer recipient** | 1.05 (0.95-1.16) |  |  |
| **Travel time to delivery facility** |  |  |  |
| <30 min. | 1.00 |  |  |
| 30 min. <1 hr. | 0.74 (0.50-1.09) |  |  |
| 1 hr. to <2 hr. | 0.94 (0.84-1.06) |  |  |
| > 2 hr. | 0.99 (0.90-1.09) |  |  |
| **Caesarean section** | 0.67 (0.45-1.01) |  |  |
| **Staff spoke your language** | 1.06 (0.96-1.18) |  |  |
| **Allowed to be accompanied?** | 0.99 (0.88-1.10) |  |  |
| **Allowed to wear clothing of choice?** | 1.13 (1.05-1.22) |  | 1.15 (1.04-1.27) |
| **Supplied bed allowing for position of choice?** | 0.98 (0.88-1.09) |  |  |
| **Allowed to consume beverage of choice** | 1.02 (0.87-1.19) |  |  |
| **Treated with respect** | 1.05 (0.85-1.29) |  |  |
| **Allowed to select the birth position** | 0.97 (0.84-1.12) |  |  |
| **HEALTH FACILITY SURVEY DATA** |  |  |  |
| **Delivery room adaptation** | 0.96 (0.88-1.05) |  |  |
| **Staff speak an indigenous language** | † |  |  |
| **Allow accompaniment when coming for delivery** | 0.98 (0.85-1.13) |  |  |
| **Allow accompaniment by community health worker** | †† |  |  |
| **Allow accompaniment by traditional birth attendant** | 1.01 (0.84-1.22) |  |  |
| **Allowable position: in a bed** | 1.18 (1.10-1.27) |  | 1.14 (1.07-1.21) |
| **Allowable position: in a chair** | 1.01 (0.87-1.16) |  |  |
| **Allowable position: on knees** | 1.18 (1.10-1.27) |  | 1.05 (1.00-1.10) |
| **Allowable position: sitting** | 1.18 (1.10-1.27) |  |  |
| **Allowable position: squatting** | 1.06 (0.82-1.39) |  |  |
| **Allowable position: standing** | †† |  |  |
| **Allowable position: vertically** | 1.18 (1.10-1.27) |  |  |

*Facility type was not assessed because all women delivered in a basic level facilities.

†Unstable estimate because more than 99.7% reported staff speaking an indigenous language.

†† Not estimable because 100% were not allowed to be accompanied by a community health worker or to delivery in a standing position.
